# Are school-based interventions to prevent dating and relationship violence and gender-based violence equally effective for all students? Systematic review and equity analysis of moderation analyses in randomised trials

**DOI:** 10.1016/j.pmedr.2023.102277

**Published:** 2023-06-08

**Authors:** G.J. Melendez-Torres, Chris Bonell, Naomi Shaw, Noreen Orr, Annah Chollet, Andrew Rizzo, Emma Rigby, Ann Hagell, Honor Young, Vashti Berry, David K. Humphreys, Caroline Farmer

**Affiliations:** a Faculty of Health and Life Sciences, University of Exeter, Exeter, UK; b Department of Public Health Environments and Society, London School of Hygiene and Tropical Medicine, London, UK; c Department of Social Policy and Intervention, University of Oxford, Oxford, UK; d College of Health and Human Performance, University of Florida, Gainesville, FL, United States; e Association for Young People’s Health, London, UK; f Centre for Development, Evaluation, Complexity and Implementation in Public Health Improvement (DECIPHer), Cardiff University, Cardiff, UK

**Keywords:** Gender-based violence, Dating and relationship violence, School health, Health equity, Systematic review, Gender inequity

## Abstract

School-based interventions for the prevention of dating and relationship violence (DRV) and gender-based violence (GBV) take advantage of universal opportunities for intervention. Information on differential effectiveness of interventions is important to assess if they ameliorate or worsen social gradients in specific outcomes. This is especially important in DRV and GBV prevention given the gendered context of these behaviours and their common aetiologies in patriarchal gender norms, and social acceptance in school contexts of sexual harassment, such as catcalling or unwanted groping. We undertook a systematic review of moderation analyses in randomised trials of school-based interventions for DRV and GBV prevention. We searched 21 databases and used supplementary search methods without regard to publication type, language or year of publication, and synthesised moderation tests relating to equity-relevant characteristics (principally sex and prior history of the outcome) for DRV and GBV perpetration and victimisation. Across 23 included outcome evaluations, programme effects on DRV victimisation were not moderated by gender or prior experience of DRV victimisation, but DRV perpetration outcomes were greater for boys, particularly for emotional and physical DRV perpetration. Findings for GBV outcomes were counterintuitive. Our findings suggest that practitioners should carefully monitor local intervention effectiveness and equity to ensure that interventions are working as intended. However, one of the most surprising findings from our analysis—with clear relevance for uncertainties in practice—was that differential impacts by sexuality or sexual minority status were not frequently evaluated.

## Highlights

• Gender-based and dating violence cause health inequities for adolescents.
• School-based interventions may not work equally well for all students.
• Moderator analyses from randomised trials suggest uneven effectiveness by sex.
• Moderators relating to other equity-relevant factors remain poorly explored.

## 1 Introduction

This paper reports a systematic review of moderator analyses from school-based interventions for the prevention of dating and relationship violence (DRV) and gender-based violence (GBV) provided to students in compulsory education (aged 5 to 18). This synthesis examines whether such interventions contribute to reducing health inequalities arising from these outcomes. DRV refers to physical, sexual and emotional violence in relationships between young people. GBV refers to violence rooted in gender and sexuality inequality (Jewkes et al., 2015). Common risk factors for DRV and GBV victimisation are well understood and experience of one predicts victimisation of the other (Exner-Cortens et al., 2013, Exner-Cortens et al., 2017) but DRV and GBV are rarely considered as joint constructs (Taquette and Monteiro, 2019). DRV and GBV are important public health problems with multiple, inequity-generating life-course health impacts. In adolescence, perpetrators and victims report increased sexual risk behaviours, substance use and depressive symptoms.(Shorey et al., 2015, Barter and Stanley, 2016, Fellmeth et al., 2013, Decker et al., 2018) In adulthood, survivors are more likely to be re-victimised (Vivolo-Kantor et al., 2016) and to report worse mental and physical health (Loxton et al., 2017). DRV and GBV experiences in adolescence predict adult experiences of domestic violence (Costa et al., 2015).

Impacts of DRV and GBV are disproportionately experienced by girls (Wado et al., 2021). Sexual violence victimisation is also more commonly reported by girls (Théorêt et al., 2021), who are also more vulnerable to the negative psychosocial sequelae of these experiences (Hébert et al., 2017). DRV and GBV can exacerbate health inequalities between men and women (Reidy et al., 2016); in particular, earlier onset of intimate partner violence leads to greater impacts on mental and physical health in adulthood (Loxton et al., 2017). Sexual-minority adolescents experience higher levels of GBV than other adolescents in terms of homophobic and transphobic bullying and sexual harassment (Decker et al., 2018, Mueller et al., 2015). Such adolescents also experience higher rates of physical and sexual DRV (Decker et al., 2018, Martin-Storey et al., 2021, Norris and Orchowski, 2020, Martin-Storey, 2015). DRV and GBV also contribute to inequalities between heterosexual and cisgender young people and their sexual-minority peers. Most notable among these inequalities are misuse of alcohol and other drugs and increased risk of suicidal ideation (Decker et al., 2018, Mueller et al., 2015). Importantly, a key source of these inequalities in mental health is the shared impact of school context, including prevalence and response to DRV and GBV, both of which point to the importance of school-based interventions (Espelage et al., 2016). There is also consistent evidence for a higher prevalence of DRV among minority ethnic groups (Mueller et al., 2015, Earnest and Brady, 2016, Coker et al., 2014, Ahonen and Loeber, 2016, Boafo et al., 2014). There is less consistent evidence that DRV and GBV are associated with individual socio-economic status or area deprivation (Wado et al., 2021, Barter et al., 2009, Hird, 2000) but some United States and South African studies do report such associations (Earnest and Brady, 2016, Coker et al., 2014, Boafo et al., 2014, Copp et al., 2015).

DRV and GBV are amenable to intervention in schools via various approaches ranging from educational (e.g. classroom teaching) to structural (e.g. school policy changes) (Jewkes et al., 2015). Systematic reviews published since 2013 have focused on DRV to the exclusion of GBV, and have not synthesised evidence of the effects of interventions on health inequalities (Fellmeth et al., 2013, De La Rue et al., 2017, Kettrey et al., 2019, Stanley et al., 2015, De Koker et al., 2014, Piolanti and Foran, 2022). Information on this is important to assess if interventions ameliorate or worsen inequalities and determine which interventions are appropriate for which populations. This is potentially important in DRV and GBV prevention given the evidence of existing inequalities in gender and other socio-demographic factors described above and the common aetiologies of DRV and GBV in patriarchal and homophobic norms at the societal level, inadequate violence-prevention policies at the school level, and individual-level exposure to and reinforcement of antisocial norms relating to gender, sexuality and violence (Taquette and Monteiro, 2019). In addition, moderation by prior experience of DRV or GBV suggests whether interventions are more effectve as primary or secondary prevention. We therefore undertook a systematic review of moderation analyses in randomised controlled trials to assess whether interventions to prevent DRV and GBV are equity-promoting. This examined whether school-based intervention effects on DRV and GBV victimisation and perpetration are moderated by ethnicity, socio-economic position, gender, sexuality and age.

## 2 Methods

This systematic review was part of a larger evidence synthesis project with a protocol registered on PROSPERO (CRD42020190463). As this was a systematic review, it did not require ethics approval.

### 2.1 Inclusion criteria

Randomised-controlled trials that evaluated moderating factors of intervention effectiveness were included, including both parallel and cluster designs. Trial populations were children of compulsory school-age (5- to 18-years of age). Interventions were included if they were implemented within school settings and were partially or wholly targeted at reducing DRV or GBV outcomes. No restriction was placed on the content of interventions or the method of delivery. Comparisons with a control intervention (including no intervention, waitlist, usual practice or an active control) were included. Analyses that investigated the moderation of DRV or GBV perpetration or victimisation outcomes were included, regardless of the moderating factor or the findings. Data from formal moderation tests as well as raw event rate data for moderator subgroups, where available, were extracted.

### 2.2 Search, selection and data extraction

A literature search was conducted in July 2020 across a broad range of bibliographic databases including Ovid MEDLINE, Embase, APA PsycINFO; EBSCO CINAHL, Education Research Complete, ERIC; ProQuest ASSIA, Sociological Abstracts, Dissertations and Theses; Web of Science Social Science Citation Index, and the Cochrane Central Register of Controlled Trials. Searches were not limited by date, language or publication type. Database searches used a combination of free-text terms and subject headings for schools and DRV/GBV. The search was updated in June 2021 with additional free-text search terms for interventions identified in the original search.

Forwards and backwards citation searching on included studies was also used, and the reference lists of relevant systematic reviews and reports were reviewed. Supplementary searches were also conducted, including targeted author name searches in Web of Science and Scopus, and searches of key websites, trial registries and Google Scholar. Search results were downloaded into EndNote X9 (Clarivate Analytics) for deduplication. Full details of the literature search strategy and sources are provided in Supplementary File 1.

Records identified in the search were screened for inclusion by two reviewers at both title/abstract and full text. Publications were not excluded at title/abstract on the basis of outcome. Disagreements were resolved through discussion and with the involvement of a third reviewer as necessary. Data were extracted into a piloted data extraction form and checked by a second reviewer.

### 2.3 Synthesis strategy

We synthesised evidence narratively, considering each test of effect modification within an outcome evaluation as a data point. We first considered whether enough studies were presented to construct a harvest plot for overall outcomes and then, where possible, explored findings by type of DRV or GBV. Harvest plots are an imaging tool used to depict the cumulative evidence of effect modification across included studies with individual bars representing trials organised by rows defined by outcomes and placed according to the moderation evidence (e.g. whether trial evidence suggests greater impact for boys, greater impact girls, or no gradient of impact). Further details are presented in Box 1. To minimise double-counting, the number of relevant interaction tests for each study and each outcome is the number of tests for non-overlapping outcome constructs; i.e. where tests are undertaken for multiple types of violence and for a violence construct that is the sum of all of these types, overall tests are not reflected in the harvest plots.

Box 1. Interpretation of harvest plots

Bars vary by height, colour and shading, and have a number that represents the number of relevant interaction tests for that moderator and outcome.

The **height** of bars represents the significance and direction of moderation presented by a trial. Full height bars represent significant moderation for a given outcome demonstrating greater intervention impact for one group. Three-quarters height bars represent a pattern of moderation estimates including some, but not consistent, significant evidence of greater intervention impact for one group. Half-height bars represent consistently non-significant estimates of moderation trending in one direction, and quarter-height bars are used for studies that only present non-significance as opposed to moderation estimates.

The **colour** of bars represents the timeframe of outcomes analysed. Blue bars represent moderation evidence from long-term outcomes, red bars represent moderation evidence from short-term outcomes, and purple bars represent moderation evidence from both short-term and long-term outcomes.

Finally, the **shading** of bars represents the nature of the moderation analysis. Dark shaded bars represent formal interaction tests, whereas light shaded bars represent outcome evaluations where formal interaction tests were not presented.

A common issue in included analyses was the lack of a formal test for moderation, either by significance of an interaction term, a Wald test or a likelihood ratio test comparing nested models. Where possible, we were able to construct statistical estimates of effect modification by using estimates from subgroups with a standard z-test for equality of means, using log-transformed estimates where necessary (e.g for effect estimates expressed as odds ratios). Failing this, we were able to approximate direction and likely significance of interaction estimates based on additional within-trial information (e.g. information on precision and sample size, and similarity of effect estimates between groups).

Given the lack of an agreed appraisal tool for moderation analyses, we did not specifically appraise moderation analyses, instead highlighting where these were imputed as a key marker of quality.

## 3 Results

A total of 26 reports of 23 outcome evaluations comparing active vs control were included in our synthesis of moderation evidence (see Supplementary File 1).

The largest category of moderator analyses covered sex, comparing boys versus girls as a binary variable (notably, no included moderation analyses considered gender as opposed to sex variation). These moderator analyses were reported in 20 outcome evaluations. Six outcome evaluations considered prior history of the outcome as a moderator (e.g. whether prior history of DRV perpetration moderates intervention impacts in reducing DRV perpetration).

Five outcome evaluations considered ethnicity as a moderator, contrasting majority populations against ethnic minority populations. A further moderator analysis (Peskin et al., 2014) from an outcome evaluation presented stratified estimates for different ethnic minority groups but, because this was a ‘majority-minority’ population, these analyses were incommensurate with other moderator analyses. Age, defined in various ways (e.g. biological age, grade level) was tested as a moderator in four outcome evaluations. Two outcome evaluations each examined dating history and sexuality as moderators of intervention impact. Acculturation (Jaycox et al., 2006) and poverty status (Waterman et al., 2022) were assessed as moderators in one outcome evaluation each.

Formal moderation tests were not presented in nine outcome evaluations (Decker et al., 2018, Peskin et al., 2014, Jaycox et al., 2006, ICRW, 2017, Coker et al., 2017, Coker et al., 2020, Foshee et al., 1998, Foshee et al., 2000, Munoz-Rivas et al., 2019, Peskin et al., 2019) comparing active versus control comparisons. Of these, we were able to construct statistical estimates of effect modification for four outcome evaluations (Decker et al., 2018, Peskin et al., 2014, Coker et al., 2017, Coker et al., 2020, Munoz-Rivas et al., 2019). For the remaining five evaluations (Jaycox et al., 2006, ICRW, 2017, Foshee et al., 1998, Foshee et al., 2000, Peskin et al., 2019), we approximated a test of effect modification from available evidence.

### 3.1 Moderation of DRV victimisation

Moderation of intervention impacts on DRV victimisation was tested in 13 outcome evaluations (Peskin et al., 2014, Jaycox et al., 2006, Coker et al., 2017, Coker et al., 2020, Foshee et al., 1998, Foshee et al., 2000, Peskin et al., 2019, Cissner and Ayoub, 2014, Foshee et al., 2004, Foshee et al., 2005, Gonzalez-Guarda et al., 2015, Joppa et al., 2016, Levesque et al., 2016, Miller et al., 2015, Muck et al., 2021, Taylor et al., 2015, Taylor et al., 2010).

#### 3.1.1 Sex as a moderator of DRV victimisation outcomes

In total, 12 outcome evaluations (Peskin et al., 2014, Jaycox et al., 2006, Coker et al., 2017, Foshee et al., 1998, Foshee et al., 2000, Cissner and Ayoub, 2014, Foshee et al., 2004, Foshee et al., 2005, Gonzalez-Guarda et al., 2015, Joppa et al., 2016, Levesque et al., 2016, Miller et al., 2015, Muck et al., 2021, Taylor et al., 2015, Taylor et al., 2010) reported moderation of DRV victimisation outcomes by sex. Two trials (Coker et al., 2017, Taylor et al., 2010) demonstrated a pattern of non-significant findings where moderation effects tended to favour boys but none of these rose to significance. An additional eight outcome evaluations (Peskin et al., 2014, Jaycox et al., 2006, Foshee et al., 1998, Foshee et al., 2000, Foshee et al., 2004, Foshee et al., 2005, Gonzalez-Guarda et al., 2015, Joppa et al., 2016, Levesque et al., 2016, Miller et al., 2015, Muck et al., 2021) did not offer evidence of a gradient for DRV victimisation outcomes. Two of these reports (Peskin et al., 2014, Gonzalez-Guarda et al., 2015) presented subgroup specific estimates but found contradictory estimates by subgroup. Six outcome evaluations (Jaycox et al., 2006, Foshee et al., 1998, Foshee et al., 2000, Foshee et al., 2004, Foshee et al., 2005, Joppa et al., 2016, Levesque et al., 2016, Miller et al., 2015, Muck et al., 2021) only reported non-significance. A final two evaluations (Cissner and Ayoub, 2014, Taylor et al., 2015) suggested greater impact for girls. One of these (Cissner and Ayoub, 2014) suggested a long-term, statistically significant gradient in favour of girls.

**Fig. 1:**
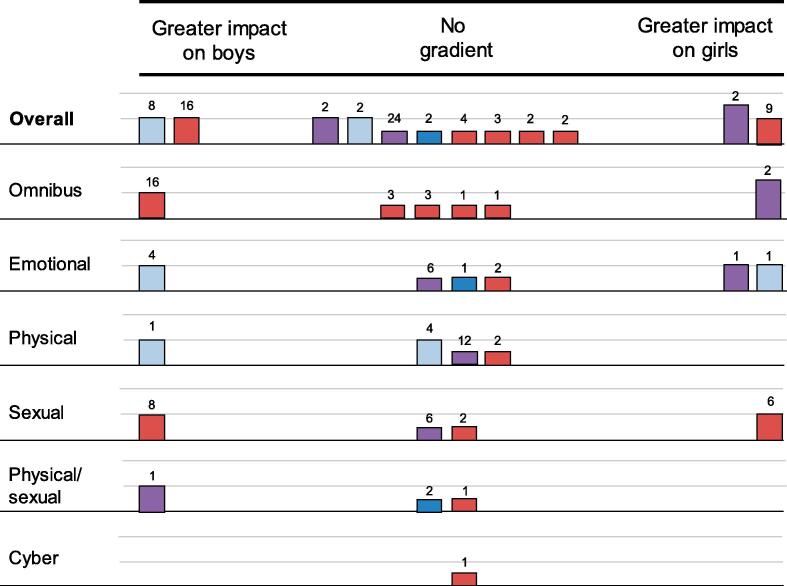
Harvest plot, DRV victimisation, boys vs girls.

The resultant harvest plot for this outcome and moderator is shown in Fig. 1. Taking all outcomes together, the harvest plot does not provide evidence for a gradient in effectiveness on DRV victimisation outcomes by sex. Because of the size of the body of evidence, we undertook an exploratory analysis stratifying outcomes by type of DRV victimisation. This did not uncover any evidence of sex-specific moderation on different types of DRV victimisation.

#### 3.1.2 Prior history of the outcome as a moderator of DRV victimisation outcomes

Five outcome evaluations (Foshee et al., 1998, Foshee et al., 2000, Levesque et al., 2016, Miller et al., 2015, Foshee et al., 2004, Foshee et al., 2005) reported prior history of DRV victimisation as a moderator of intervention effectiveness on DRV victimisation. Four evaluations suggested greater impact for those with prior history, with one (Levesque et al., 2016) yielding consistently greater evidence of this effect over the long-term, two yielding mixed findings with some significant tests (one long-term (Cissner and Ayoub, 2014) and one short-term (Miller et al, 2015)) and one (Taylor et al., 2015) yielding numerically, but not statistically, greater short-term impacts for those with prior history of DRV victimisation. One outcome evaluation (Foshee et al., 1998, Foshee et al., 2000, Foshee et al., 2004, Foshee et al., 2005) covering short-term, long-term and longitudinal analyses suggested long-term greater effectiveness for those with no prior history of DRV victimisation.

**Fig. 2:**
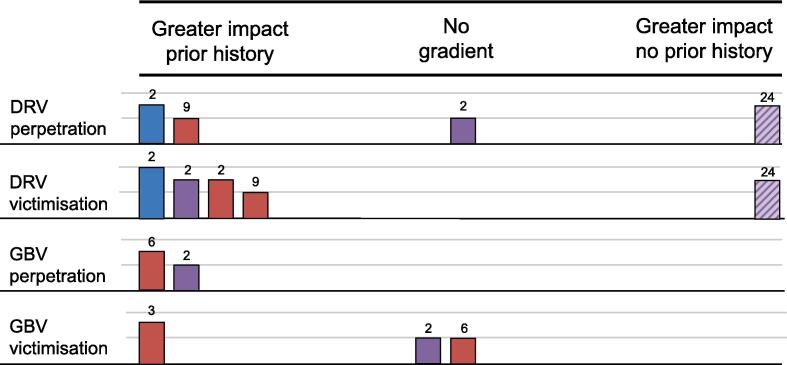
Harvest plot, prior history of the outcome, all outcomes. *Moderation by prior history of the outcome is identified when the baseline value of the outcome moderates intervention effectiveness,* e.g. *intervention effects on DRV perpetration are moderated by whether participants report DRV perpetration at baseline.*

The resultant harvest plot for this outcome and moderator is shown in Fig. 2. Taking all outcomes together, the harvest plot does not provide evidence for a gradient in effectiveness on DRV victimisation outcomes by prior history of the outcome.

### 3.2 Moderation of DRV perpetration

Moderation of intervention impacts on DRV perpetration was tested in 14 outcome evaluations (Jaycox et al., 2006, Coker et al., 2017, Coker et al., 2020, Foshee et al., 1998, Foshee et al., 2000, Munoz-Rivas et al., 2019, Peskin et al., 2019, Cissner and Ayoub, 2014, Foshee et al., 2004, Foshee et al., 2005, Gonzalez-Guarda et al., 2015, Joppa et al., 2016, Levesque et al., 2016, Muck et al., 2021, Taylor et al., 2015, Taylor et al., 2010, Wolfe et al., 2009).

#### 3.2.1 Sex as a moderator of DRV perpetration outcomes

Sex was considered as a moderator of DRV perpetration outcomes in 13 outcome evaluations (Peskin et al., 2014, Jaycox et al., 2006, Coker et al., 2017, Foshee et al., 1998, Foshee et al., 2000, Munoz-Rivas et al., 2019, Cissner and Ayoub, 2014, Foshee et al., 2004, Foshee et al., 2005, Gonzalez-Guarda et al., 2015, Joppa et al., 2016, Levesque et al., 2016, Muck et al., 2021, Taylor et al., 2015, Taylor et al., 2010, Wolfe et al., 2009). Four evaluations provided some evidence of a gradient in effectiveness favouring boys, with only one (Wolfe et al., 2009) suggesting a significant and long-term impact. Three further evaluations found non-significant evidence of a greater impact on boys over long-term (Coker et al., 2017), short-term (Taylor et al., 2015) and both short-term and long-term (Gonzalez-Guarda et al., 2015). A further seven evaluations (Peskin et al., 2014, Jaycox et al., 2006, Foshee et al., 1998, Foshee et al., 2000, Joppa et al., 2016, Levesque et al., 2016, Foshee et al., 2004, Foshee et al., 2005) did not provide evidence of a gradient in effectiveness by sex. One evaluation (Peskin et al., 2014) included two long-term tests: one of physical DRV perpetration, which numerically favoured girls, and one of emotional DRV perpetration, which numerically favoured boys. The other six evaluations (Jaycox et al., 2006, Foshee et al., 1998, Foshee et al., 2000, Joppa et al., 2016, Levesque et al., 2016, Foshee et al., 2004, Foshee et al., 2005) presented findings as non-significant only. A final two evaluations (Munoz-Rivas et al., 2019, Taylor et al., 2010) provided short-term, non-significant evidence of a greater impact on girls.

**Fig. 3:**
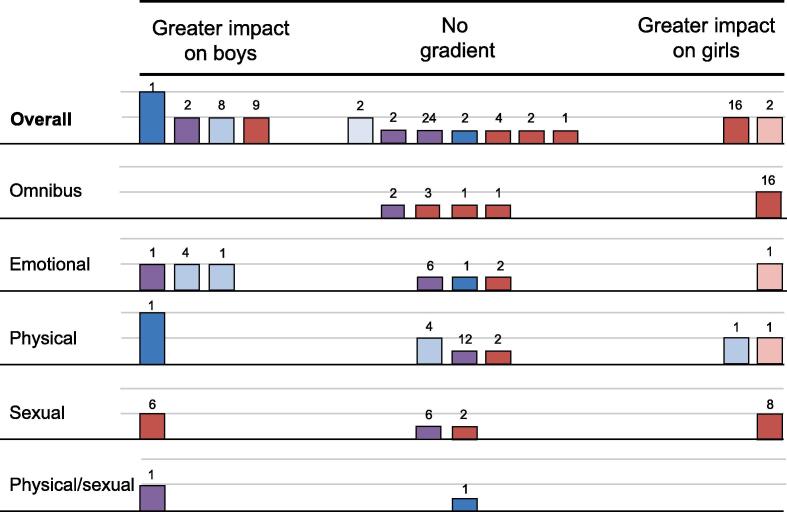
Harvest plot, DRV perpetration, boys vs girls.

The resultant harvest plot for this outcome and moderator is shown in Fig. 3. Taking all outcomes together, the harvest plot provides some evidence of a gradient in effectiveness on DRV perpetration with greater impacts for boys. This appears to be primarily driven by long-term and longitudinal evidence. Because of the size of the body of evidence, we undertook an exploratory analysis stratifying outcomes by type of DRV victimisation. This suggested that greater impacts on boys were primarily driven by evidence for emotional and physical DRV perpetration.

#### 3.2.2 Prior history of the outcome as a moderator of DRV perpetration outcomes

Four outcome evaluations (Foshee et al., 1998, Foshee et al., 2000, Levesque et al., 2016, Foshee et al., 2004, Foshee et al., 2005) considered prior history of DRV perpetration as a moderator of DRV perpetration outcomes. Two of these reports provided some evidence of a gradient with greater effects among those with prior history of DRV perpetration, with some significant long-term evidence from one evaluation (Levesque et al., 2016) and numerical, but not statistical, findings in the short-term from another one (Taylor et al., 2015). Another evaluation yielded mixed but non-significant evidence over short-term and long-term (Cissner and Ayoub, 2014). Finally, evidence from one evaluation included short-term (Foshee et al., 1998), long-term (Foshee et al., 2000, Foshee et al., 2004) and longitudinal (Foshee et al., 2005) tests found some greater benefits for those with no prior history in the long-term. The resultant harvest plot is displayed in Fig. 2.

### 3.3 Moderation of GBV victimisation

Moderation of intervention impacts on GBV victimisation was tested in 13 outcome evaluations (Decker et al., 2018, Cissner and Ayoub, 2014, Waterman et al., 2022, ICRW, 2017, Coker et al., 2017, Coker et al., 2020, Muck et al., 2021, Taylor et al., 2015, Taylor et al., 2010, Rowe et al., 2015, Jemmott et al., 2018, Devries et al., 2017, de Lijster et al., 2016).

#### 3.3.1 Sex as a moderator of GBV victimisation outcomes

Sex as a moderator of GBV victimisation outcomes was considered in 11 outcome evaluations (Cissner and Ayoub, 2014, Waterman et al., 2022, ICRW, 2017, Coker et al., 2017, Muck et al., 2021, Taylor et al., 2015, Taylor et al., 2010, Jemmott et al., 2018, Devries et al., 2017, de Lijster et al., 2016). Four outcome evaluations (ICRW, 2017, Taylor et al., 2010, Jemmott et al., 2018, Devries et al., 2017) suggested a greater impact for boys. Two long-term evaluations with formal (Jemmott et al., 2018) and informal (ICRW, 2017) tests of effect modification suggested statistically greater impacts for boys. A further two evaluations, one long-term (Devries et al., Oct 2017) and one short-term (Taylor et al., 2010) found numerical, but not statistical, evidence of greater impacts for boys.

A further five evaluations (Waterman et al., 2022, Coker et al., 2017, Cissner and Ayoub, 2014, Muck et al., 2021, de Lijster et al., 2016) did not offer evidence of a gradient in effectiveness by sex. One indicated this via subgroup analyses covering long-term timepoints (Coker et al., 2017). A subsequent four evaluations (Waterman et al., 2022, Cissner and Ayoub, 2014, Muck et al., 2021, de Lijster et al., 2016) only presented evidence of non-significance.

Finally, two evaluations suggested a greater impact on girls. One outcome evaluation (ICRW, 2017) suggested long-term, statistically greater impacts on girls. A second found numerical but not statistical evidence of greater benefits for girls (Taylor et al., 2015).

**Fig. 4:**
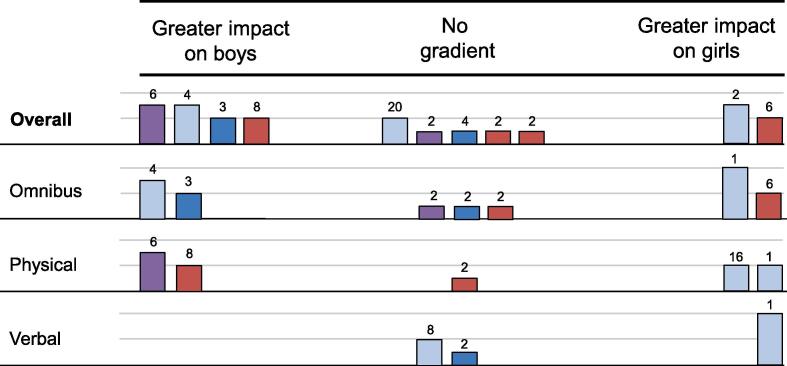
Harvest plot, GBV victimisation, boys vs girls.

The resultant harvest plot for this outcome and moderator can be seen in Fig. 4. On balance, the harvest plot suggests the plausibility of a gradient in intervention effects favouring boys, driven primarily by longer-term evidence. An exploratory analysis stratifying outcomes by type of GBV victimisation did not reveal a clear pattern explaining this distribution of study-level moderation findings, though longer-term evidence from omnibus measures of GBV victimisation did appear to favour greater impact on boys.

#### 3.3.2 Prior history of the outcome as a moderator of GBV victimisation outcomes

Three evaluations (Cissner and Ayoub, 2014, Taylor et al., 2015, Rowe et al., 2015) considered prior history of GBV victimisation as a moderator of intervention outcomes. Two evaluations were largely equivocal. One short-term evaluation (Taylor et al., 2015) and one short-term and long-term evaluation (Cissner and Ayoub, 2014) found conflicting results over different measures and timepoints, though at no point were findings significant. A final evaluation was delivered only to girls and included two short-term tests of physical GBV victimisation and one short-term test of verbal GBV victimisation. Both tests of physical GBV victimisation were non-significant but indicated greater impact for those with no prior history of GBV victimisation, while the test verbal GBV victimisation was significant and indicated greater impacts for those with prior history. The resultant harvest plot is depicted in Fig. 2.

### 3.4 Moderation of GBV perpetration

Moderation of intervention impacts on GBV perpetration was tested in 10 outcome evaluations (Cissner and Ayoub, 2014, Jemmott et al., 2018, ICRW, 2017, Coker et al., 2017, Coker et al., 2020, Muck et al., 2021, Taylor et al., 2015, Taylor et al., 2010).

#### 3.4.1 Sex as a moderator of GBV perpetration outcomes

Ten outcome evaluations (Cissner and Ayoub, 2014, Jemmott et al., 2018, ICRW, 2017, Coker et al., 2017, Muck et al., 2021, Taylor et al., 2015, Taylor et al., 2010) considered sex as a moderator of GBV perpetration outcomes. One report of an intervention in South Africa (Jemmott et al., 2018) considered physical GBV perpetration over short-term and long-term timeframes, with a collectively greater impact on boys. A further four outcome evaluations (Waterman et al., 2022, Cissner and Ayoub, 2014, Muck et al., 2021, de Lijster et al., 2016) reported only that results were not significant: one (Cissner and Ayoub, 2014) including a short-term and a long-term test of an omnibus measure of GBV perpetration, one (Waterman et al., 2022) including four long-term tests of an omnibus measure of GBV perpetration and of verbal GBV perpetration, one (Muck et al., 2021) including two short-term tests of physical GBV perpetration, and one (de Lijster et al., 2016) including one short-term test of an omnibus measure of GBV perpetration. A final set of five outcome evaluations (ICRW, 2017, Coker et al., 2017, Taylor et al., 2015, Taylor et al., 2010) showed a pattern of greater effects for girls, over short-term and long-term timepoints and a range of types of GBV. However, in every case, tests were non-significant.

**Fig. 5:**
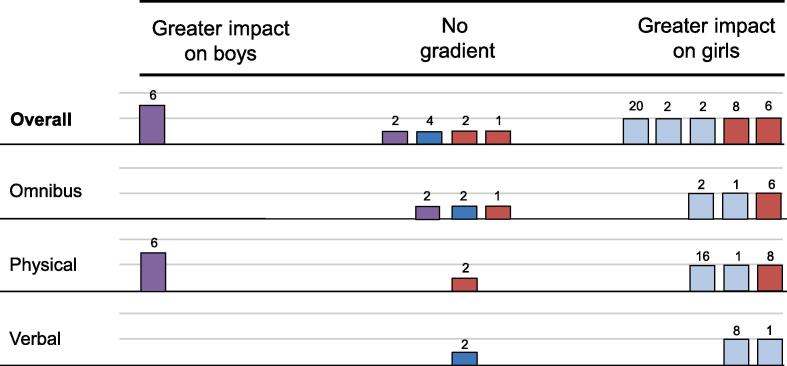
Harvest plot, GBV perpetration, boys vs girls.

The resultant harvest plot is displayed in Fig. 5. A plausible interpretation of the collected evidence is that interventions may be more effective for girls than for boys, though this is countervailed by the evidence from one evaluation (Jemmott et al., 2018) featuring short-term and long-term follow-up and the relatively high proportion of evaluations reporting non-significance only. As an exploratory analysis, we stratified outcomes by type of GBV victimisation. This suggests that evidence for a gradient favouring greater impact on girls is strongest for omnibus measures and verbal GBV perpetration, albeit with relatively few studies supporting each conclusion.

#### 3.4.2 Prior history of the outcome as a moderator of GBV perpetration outcomes

Two evaluations (Cissner and Ayoub, 2014, Taylor et al., 2015) considered prior history of GBV perpetration as a moderator of intervention outcomes. Both collectively indicated that interventions could be more effective for students with a prior history of GBV perpetration. The resultant harvest plot is depicted in Fig. 2.

### 3.5 Additional moderators

Moderators pertaining to dating history, age, ethnicity, acculturation, sexuality, and poverty status are presented in Supplementary File 2. No evidence of moderation was revealed in presented analyses. In addition, restricting our analyses to studies reporting in the last decade did not change our conclusions.

## 4 Discussion

The majority of equity-relevant analyses reported moderation related to sex and prior history of the outcome. All other evidence for equity-relevant moderators, including age, ethnicity, sexuality and poverty, were sparse and did not present conclusive indication of moderation within outcome. Our syntheses suggested that programme effects on DRV victimisation were not moderated by gender or prior experience of DRV victimisation. However, there was evidence from multiple studies for gender moderating programme effects on DRV perpetration with greater benefits for boys, particularly for emotional and physical DRV perpetration. There was weaker evidence that programme effects were greater for those with prior experience of DRV perpetration.

In contrast to DRV victimisation, there was some evidence that programmes had greater effects reducing GBV victimisation among boys than girls, driven primarily by longer-term evidence. Two studies examined prior experience of GBV victimisation finding little evidence of moderation. The finding of a larger effect for male than female GBV victimisation is surprising and hard to interpret. Similarly, there was some, albeit patchy, evidence that programmes were more effective in reducing GBV perpetration for girls than for boys, and some evidence that programmes were more effective for those with prior experience of GBV perpetration.

### 4.1 Implications for research, policy and practice

The finding that, for some DRV perpetration outcomes, effects were larger for boys suggests that these programmes were not gender-neutral in their impacts and, possibly, in their delivery and mechanisms of impact. The programmes may have been interpreted by students as programmes aiming to reduce male perpetration of DRV and, also informed by the above findings on mediation, might have achieved these effects via changes in male attitudes to violence. There was weak evidence that such mechanisms might have been slightly stronger among those who had previously engaged in perpetration. The finding that programmes may have been more effective in reducing GBV perpetration among girls more than boys is unexpected and not easily interpreted. It might possibly be that programmes encouraging girls not to perpetrate GBV were more novel and therefore impactful than similar messages concerning male perpetration, or that interventions may be more effective when targeting audiences regarding GBV as a less acceptable behaviour. However, this is speculative and this finding adds to the overall picture that GBV programmes may not work as theorised; i.e. with respect to gendered theories of violence.

From a practice perspective, our findings suggest that interventions are just as, if not more, effective as secondary prevention compared to primary prevention. Our findings also suggest that practitioners should carefully monitor local intervention effectiveness and equity to ensure that interventions are working as intended. However, one of the most surprising findings from our analysis—with clear relevance for uncertainties in practice—was that differential impacts by sexuality or sexual minority status were not frequently evaluated. This is despite the substantial burden of GBV that sexual minority young people experience, including as regards homophobic and transphobic violence. In addition, age-related moderation analyses were scant, despite their potential value in identifying a ‘critical period’ for DRV and GBV prevention, ideally building on early years learning about healthy relationships and social skills.

### 4.2 Strengths and limitations

A strength of this analysis is the extensive search and the comprehensive presentation of included moderator analyses. This is the first review of its kind in this area. However, as with any review, it is possible that some relevant studies were missed. In addition, as noted above, not all studies presented complete information on moderation analyses; and it is possible, if not likely, that some exploratory analyses were not reported. This is especially the case if findings were not significant or otherwise judged uninteresting. These possibilities cannot be excluded, nor their impact on the findings estimated. Indeed, a challenge with harvest plots is that their ability to depict studies with moderation analyses described as ‘not significant’ is limited, given that in standard *meta*-analysis even non-significant estimates can contribute towards the overall magnitude of the effect. Finally, understanding of DRV and GBV has evolved considerably over the last decade. While this may have been reflected in changes to measuring effect modification, we did not find that removing studies reporting over a decade prior to the search affected our conclusions.

## 5 Conclusion

Our systematic review has suggested that while intervention effects for DRV perpetration are not gender-neutral, intervention effects for GBV outcomes are counterintuitive. To ensure interventions are equity-promoting, future triallists and developers should consider where and why equity effects may arise.

## Funding

This study is funded by the NIHR Public Health Research Programme (NIHR130144). In addition, Vashti Berry and G.J. Melendez-Torres are part-supported by the NIHR Applied Research Collaboration South West Peninsula (NIHR PenARC) and Chris Bonell is part-funded by an NIHR senior investigator award. The views expressed are those of the authors and not necessarily those of the NIHR or the Department of Health and Social Care. The funders had no role in the design and conduct of the study.

## Declaration of Competing Interest

The authors declare that they have no known competing financial interests or personal relationships that could have appeared to influence the work reported in this paper.

## Data Availability

Data will be made available on request.
