# Supplementary material for: Are school-based interventions to prevent dating and relationship violence and gender-based violence equally effective for all students? Systematic review and equity analysis of moderation analyses in randomised trials
Source: Prev Med Rep. 2023 Jun 8;34:102277. doi: 10.1016/j.pmedr.2023.102277 (PMC10302154; doi:10.1016/j.pmedr.2023.102277)
Supplement: Supplementary data 1 [file mmc1.docx]

# **Supplementary File 1.** Literature search strategy and data extraction

## Literature search strategy

A single search strategy was used to identify a broad range of study designs included in a large NIHR-funded systematic review of school-based interventions for DRV and GBV. A PRISMA diagram showing the source and flow of records identified in the search is provided in this document.

### Bibliographic database searches

The following bibliographic databases were searched in June 2020 and updated in June 2021:

- MEDLINE, Embase, PsycINFO, Social Policy and Practice (Ovid);
- CINAHL, ERIC, British Education Index, Education Research Complete, EconLit, Criminal Justice Abstracts (EBSCO);
- Cochrane Database of Systematic Reviews (CDSR) and the Cochrane Central Register of Controlled Trials (CENTRAL);
- NHS Economic Evaluation Database (NHS EED via the Centre for Reviews and Dissemination);
- Social Science Citation Index and Conference Proceedings Citation Index (Web of Science, Clarivate Analytics);
- Australian Education Index, ProQuest Dissertations & Theses Global, Sociological Abstracts including Social Services Abstracts, Applied Social Sciences Index and Abstracts (ProQuest);
- Trials Register of Promoting Health Interventions (TRoPHI) and Bibliomap (EPPI-Centre);
- Campbell Systematic Reviews (Campbell Collaboration).

The search strategies were developed by an experienced Information Specialist following extensive scoping searches and consideration of existing systematic reviews. The search terms used included a combination of free-text terms and subject headings for topic areas of interest. No study design filters were applied, and searches were not restricted by date or language of publication. The bibliographic database searches were revised to increase precision for the update search in June 2021. The revised strategy was based on analysis of titles, abstracts and index terms of included studies. The updated search strategy also incorporated programme names not identified in the initial scoping searches. Results from the update searches were not date limited, but instead de-duplicated against previous result sets to ensure records were not missed. An example database search strategy (for Ovid MEDLINE) is provided below.

Ovid MEDLINE search strategy (June 2020)

1 exp Intimate Partner Violence/ (9469)

2 Gender-Based Violence/ (188)

3 Stalking/ (203)

4 Rape/ (6264)

5 Sex Offenses/ (8867)

6 Battered Women/ (2620)

7 Spouse abuse/ (7352)

8 Coercion/ (4594)

9 Domestic violence/ (6375)

10 Homophobia/ (518)

11 (stalking or stalker*).ti,ab. (792)

12 rape*.ti,ab. (11749)

13 "intimate partner violence".ti,ab. (7375)

14 IPV.ti,ab. (6263)

15 (gender* adj3 violen*).ti,ab. (1623)

16 GBV.ti,ab. (1120)

17 SRGBV.ti,ab. (1)

18 (domestic adj3 (abuse* or abusive or aggressi* or assault* or attack* or bully* or coerc* or cyberbully* or femicid* or harass* or homicid* or injur* or manipulate* or murder* or rape* or threaten* or violen* or victimi?ation or revictimi?ation or re-victimi?ation)).ti,ab. (6880)

19 "violence against women".ti,ab. (2369)

20 ((date or dating) adj3 (abuse* or abusive or aggressi* or assault* or attack* or bully* or coerc* or cyberbully* or femicid* or harass* or homicid* or injur* or manipulate* or murder* or rape* or threaten* or violen* or victimi?ation or revictimi?ation or re-victimi?ation)).ti,ab. (2532)

21 ((relationship* or partner* or acquaintance* or non-stranger* or nonstranger*) adj3 (abuse* or abusive or aggressi* or assault* or attack* or bully* or coerc* or cyberbully* or femicid* or harass* or homicid* or injur* or manipulate* or murder* or rape* or threaten* or violen* or victimi?ation or revictimi?ation or re-victimi?ation)).ti,ab. (17167)

22 ((boyfriend* or boy-friend* or girlfriend* or girl-friend*) adj3 (abuse* or abusive or aggressi* or assault* or attack* or bully* or coerc* or cyberbully* or femicid* or harass* or homicid* or injur* or manipulate* or murder* or rape* or threaten* or violen* or victimi?ation or revictimi?ation or re-victimi?ation)).ti,ab. (47)

23 (interpersonal adj3 (abuse* or abusive or aggressi* or assault* or attack* or bully* or coerc* or cyberbully* or femicid* or harass* or homicid* or injur* or manipulate* or murder* or rape* or threaten* or violen* or victimi?ation or revictimi?ation or re-victimi?ation)).ti,ab. (2416)

24 (sexual* adj3 (abusive or aggressi* or assault* or attack* or bully* or coerc* or cyberbully* or femicid* or harass* or homicid* or injur* or manipulate* or murder* or rape* or threaten* or violen* or victimi?ation or revictimi?ation or re-victimi?ation)).ti,ab. (15390)

25 ((coerc* or forced or unwanted or nonconsensual or non-consensual) adj2 sex*).ti,ab. (2147)

26 (grope or groped or groping).ti,ab. (144)

27 (sext or sexts or sexting).ti,ab. (206)

28 (homophobi* or transphobi* or biphobi* or homonegativ*).ti,ab. (1662)

29 ((LGB or LGBT* or homosexual* or lesbian* or gay or bisexual* or queer* or transgender* or transsexual*) adj3 (abuse* or abusive or aggressi* or assault* or attack* or bully* or coerc* or cyberbully* or femicid* or harass* or homicid* or injur* or manipulate* or murder* or rape* or threaten* or violen* or victimi?ation or revictimi?ation or re-victimi?ation)).ti,ab. (697)

30 "long live love".ti,ab. (4)

31 (greendot or "green dot").ti,ab. (28)

32 "project respect".ti,ab. (27)

33 ("Media Aware" or mediaaware).ti,ab. (5)

34 TakeCARE.ti,ab. (14)

35 "Fourth R".ti,ab. (24)

36 "Safe Dates".ti,ab. (22)

37 "Shifting boundaries".ti,ab. (49)

38 "Teen choices".ti,ab. (4)

39 "good schools toolkit".ti,ab. (2)

40 "mentors in violence prevention".ti,ab. (5)

41 "Expect Respect".ti,ab. (8)

42 "Second Step".ti,ab. (10890)

43 SS-SSTP.ti,ab. (1)

44 "It's your game".ti,ab. (12)

45 DaVIPoP.ti,ab. (0)

46 (Benzies adj2 Batchies).ti,ab. (1)

47 or/1-46 (80507)

48 Schools/ (37880)

49 exp School Health Services/ (22957)

50 Students/ (58229)

51 Curriculum/ (74944)

52 school*.ti,ab,jw. (290890)

53 (pupil or pupils).ti,ab. (21829)

54 (classroom* or class-room*).ti,ab. (17106)

55 or/48-54 (420474)

56 47 and 55 (5600)

### Additional searches

We searched trial registers to identify ongoing or unpublished research (clinicaltrials.gov, WHO ICTRP) and we conducted searches for grey literature including conference abstracts, reports and theses from web searches, repositories of grey literature (e.g. OpenGrey.eu) as well as searches of websites identified in initial scoping searches (including VAWnet; [www.vawnet.org](http://www.vawnet.org)).

In addition, the reference lists of existing systematic reviews or relevant reports were reviewed for relevant literature. Forward and backward citation chasing was conducted on included studies identified from the June 2020 bibliographic database searches. Scopus (Elsevier), Web of Science (Clarivate) and Google Scholar were used for citation chasing, and bibliographies of included studies were manually checked where this information was incomplete on Web of Science and Scopus.

Targeted searches were conducted in Web of Science and Scopus using first and last author names for studies identified in bibliographic database searches in June 2020. Specific project names (for e.g. Project Respect, Shifting Boundaries or Safe Dates) were included in the update search strategies in bibliographic databases, and Google Scholar. Results were screened in Google Scholar, with the first 200 records scanned for each search string. Websites identified in initial scoping searches were browsed or searched for additional reports (including VAWnet: [www.vawnet.org](http://www.vawnet.org); USAID: [www.usaid.gov](http://www.usaid.gov); AVA – Against Violence and Abuse; UNGEI, National Criminal Justice Reference Service: [www.ncjrs.gov](http://www.ncjrs.gov)).

## PRISMA diagram

Records identified from database searching (n=54,064)

Duplicate records removed before screening

(n=28,048)

Records screened at title/abstract

(n=26,016)

Records excluded

(n=25,517)

Reports sought for retrieval

(n=499)

Reports not retrieved

(n=1)

Reports assessed for eligibility (n=498)

searching)

Reports excluded (n=369)

Studies included in review:

outcome evaluations (n=68); process evaluations (n=137);

economic evaluations (n=0);

costing and resource use (n=7);

mediation analyses (n=5)

moderation analyses (n=24)

from total reports (n=247)

Records identified from:

- Website searches (n=10)
- Google Scholar searches (n=26)
- Citation chasing (n=18,571)
- Reference lists of existing systematic reviews (n=134)
- First-last author searching (n=18,941)

Records screened at title/abstract

(n= 14,144)

Records excluded (n=13,838)

c

Reports sought for retrieval

(n=306)

Reports not retrieved

(n=16)

Reports assessed for eligibility

(n=290)

Reports excluded (n=172)

Moderation analyses included in this report (comparisons with control interventions; n=23)

# Overview of included studies

| **Author, date** | **Location** | **Enrolment or data collection** | **Sample size** | **Trial arms (clusters)** | **Age range (mean, SD)** | **% Female** | **Comparison** | **Moderation analyses (presented vs imputed)** |
| --- | --- | --- | --- | --- | --- | --- | --- | --- |
| Cissner 2014 | USA, North America | 2011 - 2012 | 1577 | 2 (NR, approx. 90) | 11 – 14 (12, NR) | 55.2 | Stay Strong Bronx vs. no intervention | **Presented**  Girls vs. boys  Prior history at baseline |
| Coker 2017, 2020 | USA, North America | 2010 - 2014 | 89707 | 2 (26) | Grade 9 - Grade 12 (NR) | 54.1 - 54.6 | Green Dot vs no intervention | **Imputed**  Girls vs. boys  Sexuality |
| De Lijster 2016 | The Netherlands, Europe |  | 815 | 2 (28) | NR (14.62 – 14.4, 0.70 – 0.82) | 51 | Benzies & Batchies vs. wait list | **Presented**  Girls vs. boys  Ethnicity  Educational level  Age |
| Decker 2018 | Malawi, Africa | 2015 | 5199 | 2 (151) | NR (15.28, 2.06) | 100 | IMPower vs. no intervention | **Imputed**  Age |
| Devries 2017 | Uganda, Africa | 2012 - 2014 | 3820 | 2 (42) | NR (13.0, 1.5) | 52.3 | The Good School Toolkit vs. Waitlist | **Presented**  Girls vs. boys |
| Foshee 1998, 2000, 2004, 2005 | USA, North America | 1994 - 1995 | 2344 | 2 (NA) | 11 - 17 (13.8, NR) | 51.1 | Safe Dates vs. No intervention (+ community intervention) | **Presented**  Girls vs. boys  Ethnicity  **Presented/imputed**  Prior history at baseline  **Imputed**  Dating history |
| Gonzalez-Guarda 2015 | USA, North America | 2012 - 2013 | 82 | 2 (NA) | 13 - 16 (14.34, 0.65) | 56 | JOVEN vs waitlist | **Presented**  Girls vs. boys |
| ICRW 2017_India | India, Asia | 2014 - 2016 | 4000 |  | 10 – 15 (NR) | 55.3 | GEMS vs. no intervention | **Imputed**  Girls vs. boys |
| ICRW 2017_Vietnam | Vietnam, Asia | 2012 - 2015 | 816 |  | 12 – 14 (NR) | 50 | GEMS vs. no intervention | **Imputed**  Girls vs. boys |
| Jaycox 2006 | USA, North America | 2001 - 2004 | 2617 | 2 (40) | NR (14.41, 1.02) | 51.7 | Ending violence vs usual practice (health classes) | **Presented**  Girls vs. boys  **Presented/imputed**  Acculturation (English proficiency) |
| Jemmott 2018 | South Africa, Africa | 2004 - 2005 | 1118 | 2 (18) | 9 - 18 (12.4, 1.2) | 53 | HIV/STD risk-reduction intervention vs. active control | **Presented**  Girls vs. boys |
| Joppa 2016 | USA, North America | 2013 - 2013 | 598 | 2 (24) | 14 - 19 (15.85, 0.85) | 54 | Katie Brown Educational Program vs. waitlist | **Presented**  Girls vs. boys  Ethnicity |
| Levesque 2016 | USA, North America | 2009 - 2009 | 3901 | 2 (20) | NR (NR) | 51.7 | Teen choices vs active control ('health in motion') | **Presented**  Girls vs. boys  Prior history at baseline  Ethnicity  Age |
| Miller 2015 | USA, North America | 2012 | 1012 | 2 (10) | 14 - 19 (NR) | 76.3 | School Health Center Healthy Adolescent Relationships Program (SHARP) vs. usual practice | **Presented**  Girls vs. boys  Prior history at baseline |
| Muck 2018a | Germany, Europe | 2014 - 2015 | 453 | 3 (27) | 12 - 16 (14.18, 0.71) | 55 | Scientist-Practitioner Program vs. Practitioner Program vs. control | **Presented**  Girls vs. boys |
| Munoz-Rivas 2019 | Spain, Europe | NR | 841 | 2 (45) | 14 - 17 (14.63, 0.69) | 60.5 | PREVIO vs no intervention | **Imputed**  Girls vs. boys |
| Peskin 2014 | USA, North America | 2004 | 1445 | 2 (10) | NR (13.0, 0.54) | 57.8 | It's your game…keep it real vs. usual practice | **Imputed**  Girls vs. boys |
| Peskin 2019 | USA, North America | 2014 | 1760 | 2 (10) | 11.17 - 14.55 (12.2, 0.59) | 52.5 | Me and You vs. usual practice | **Imputed**  Dating history |
| Rowe 2015 | USA, North America | NR | 85 | 2 (NA) | 14 - 18 (15.63, NR) | 100 | My voice, my choice vs. no intervention | **Presented**  Prior history at baseline |
| Taylor 2010 | USA, North America | 2006 - 2007 | 1639 | 3 (123) | 11 - 13 (NR) | 52 | Interaction curriculum vs law and justice curriculum vs control | **Presented**  Girls vs. boys |
| Taylor 2015 | USA, North America | 2009 - 2010 | 2665 | 4 (117) | 10 - 15 (NR) | 53 | Shifting boundaries: Building + classroom vs building only vs classroom only vs control group | **Presented**  Girls vs. boys  Prior history at baseline |
| Waterman 2021 (based on Edwards 2019) | USA, North America | NR | 2403 | 2 (25) | 13 - 19 (15.8, 1.2) | 50.9 | Bringing in the Bystander—High School Curriculum [BITB-HSC] vs. no intervention | **Presented**  Girls vs. boys  Ethnicity  Age  Poverty status  Sexuality |
| Wolfe 2009 | USA, North America | 2003 | 1722 | 2 (20) | 14 - 15 (NR) | 52.8 | Fourth R: Skills for Youth Relationships vs usual practice | **Presented**  Girls vs. boys |
