# Supplementary material for: Are school-based interventions to prevent dating and relationship violence and gender-based violence equally effective for all students? Systematic review and equity analysis of moderation analyses in randomised trials
Source: Prev Med Rep. 2023 Jun 8;34:102277. doi: 10.1016/j.pmedr.2023.102277 (PMC10302154; doi:10.1016/j.pmedr.2023.102277)
Supplement: Supplementary data 3 [file mmc3.docx]

# **Supplementary File 2.** Detailed syntheses and additional moderation analyses

*Sex as a moderator of DRV victimisation outcomes*

Two trials(1, 2) demonstrated a pattern of non-significant findings where moderation effects tended to favour boys. An analysis of Green Dot(1) presented evidence stratified by sex for long-term findings for physical and emotional DRV victimisation; we calculated interaction effects and did not find that the prevalence risk ratio for any outcome measure was significantly different between boys and girls, though the pattern of effects on balance favoured boys (five out of eight interaction tests). An analysis of subgroup effects from the Cleveland trial of law and justice and interaction-based programmes(2) drew on short-term evidence and included 24 tests, of which 16 were ‘unique’, covering omnibus measures of DRV victimisation and sexual DRV victimisation. No interaction tests were significant, but 18 of 24 tests (12 of 16 unique tests) suggested a greater impact on boys.

An additional eight outcome evaluations(3-13) did not offer evidence of a gradient for DRV victimisation outcomes. Two of these reports(7, 13) presented subgroup specific estimates. In the evaluation of JOVEN,(7) neither test of moderation over both short-term and long-term time categories yielded a significant result; however, stratified evidence suggested a greater impact of the intervention for girls on emotional DRV victimisation and a greater impact for boys on physical and sexual DRV victimisation. Six outcome evaluations only reported significance.(3-6, 8-12) Moderation analyses of Safe Dates were presented in four reports(3-6) covering short-term,(3) long-term,(4, 6) and longitudinal(5) (both short-term and long-term) analyses of outcomes, representing 24 tests in total. In all reports, moderation tests were presented for physical DRV victimisation, sexual DRV victimisation and emotional DRV victimisation and all were reported as non-significant. A moderation test of Teen Choices(10) for long-term physical and sexual DRV victimisation and emotional DRV victimisation also reported both tests as non-significant. The remaining four evaluations all presented evidence in respect of short-term outcomes. Moderation tests for the Katie Brown Educational Program(9) included five tests, of which four were unique, covering an omnibus measure of DRV victimisation, emotional DRV victimisation and physical DRV victimisation. All tests were reported as non-significant. Three tests of moderation in the evaluation of Ending Violence,(8) all of which were omnibus measures of DRV victimisation, were reported as non-significant. Three tests of moderation (two unique) in the evaluation of the School Health Center Healthy Adolescent Relationships Program (SHARP)(11) related to cyber DRV victimisation, physical and sexual DRV victimisation, and an omnibus measure of DRV victimisation. All were reported as non-significant. Lastly, an evaluation of two programmes(12) against a control condition yielded two tests of sexual DRV victimisation, reporting non-significant results.

A final two evaluations(14, 15) suggested greater impact for girls. The New York City evaluation of Fourth R(14) included two tests of moderation of omnibus measures of DRV victimisation. A short-term analysis yielded a numerical, but not significant, estimate of greater impact for boys, but a long-term analysis yielded a significant test (*p*<0.05) suggesting greater impact for girls. Finally, the moderation analysis for Shifting Boundaries included nine tests covering omnibus measures of DRV victimisation and sexual DRV victimisation. While all nine tests were reported as non-significant, five of six tests of sexual DRV victimisation suggested a numerically greater impact for girls. All three tests of omnibus measures were reported as non-significant only.

Prior history of the outcome as a moderator of DRV victimisation outcomes

The evaluation of Teen Choices(10) included two tests of long-term outcomes relating to physical and sexual DRV victimisation and emotional DRV victimisation. Both tests were significant at *p*<0.01 level, reflecting a substantially greater impact for those with prior history of DRV victimisation in reducing future DRV victimisation. The New York City evaluation of Fourth R(14) included two tests of moderation of omnibus measures of DRV victimisation. A short-term analysis yielded a numerical, but not significant, estimate of greater impact for those with no prior history of the outcome, but a long-term analysis yielded a significant test (*p*<0.05) suggesting greater impact for those with prior history. Three tests of moderation (two unique) in the evaluation of the School Health Center Healthy Adolescent Relationships Program (SHARP)(11) related to cyber DRV victimisation, physical and sexual DRV victimisation, and an omnibus measure of DRV victimisation. All favoured those with prior history of DRV victimisation, with both the cyber DRV victimisation and the omnibus measure generating significant tests. Lastly, the evaluation of Shifting Boundaries(15) included nine tests covering omnibus measures of DRV victimisation and sexual DRV victimisation. While all nine tests were reported as non-significant, four of six tests of sexual DRV victimisation suggested a numerically greater impact for those with prior history of DRV victimisation. All three tests of omnibus measures were reported as non-significant only.

As noted above, moderation analyses of Safe Dates were presented in four reports(3-6) covering short-term,(3) long-term,(4, 6) and longitudinal(5) (both short-term and long-term) analyses of outcomes, representing 24 tests in total. As for sex as a moderator, in all reports, moderation tests were presented for physical DRV victimisation, sexual DRV victimisation and emotional DRV victimisation. In short-term and longitudinal tests,(3, 5) all results were reported as non-significant. However, long-term effects(6) at one year post-intervention were all imputed as non-significant, but favouring those with no prior history of DRV victimisation (four tests), while five out of eight tests at four years post-intervention resulted in significant estimates demonstrating greater effectiveness for those with no prior history of DRV victimisation.

*Dating history as a moderator of DRV victimisation outcomes*

Two outcome evaluations(3, 6, 16) reported dating history as a moderator of DRV victimisation outcomes; in no report was moderation tested formally. Moderation by dating history was considered in short-term(3) and long-term(6) analyses of Safe Dates. All four short-term outcomes, covering physical DRV victimisation, sexual DRV victimisation and emotional DRV victimisation, were imputed as generating non-significant moderation. Of four long-term outcomes, three of four (physical DRV victimisation and sexual DRV victimisation) appeared to generate numerically but not statistically greater impacts on those with baseline dating history. The long-term evaluation of It’s Your Game(16) compared estimates for a subsample of those with dating history at baseline to the whole sample for six outcomes (of which five were unique), covering emotional, physical, sexual, and cyber DRV victimisation as well as an omnibus measure. Based on estimates of precision, there was no evidence of significant moderation, but all six outcomes showed attenuated effects for those with prior dating history, with the exception of cyber DRV.

*Age as a moderator of DRV victimisation outcomes*

The long-term evaluation(10) of Teen Choices evaluated grade level as a moderator of effects on physical DRV victimisation and emotional DRV victimisation. In both cases, results were reported as non-significant only.

*Ethnicity as a moderator of DRV victimisation outcomes*

Three outcome evaluations(4-6, 9, 10) considered ethnicity (White vs other groups) as a moderator of DRV victimisation outcomes; all were reported as non-significant only. Ethnicity was considered as a moderator of DRV victimisation outcomes in the long-term(4, 6) and longitudinal(5) evaluations of Safe Dates, across 20 tests covering emotional DRV victimisation, physical DRV victimisation and sexual DRV victimisation. The long-term evaluation(10) of Teen Choices evaluated ethnicity as a moderator of effects on physical DRV victimisation and emotional DRV victimisation. Finally, the short-term evaluation of the Katie Brown Educational Program(9) evaluated five outcomes (four unique) covering an omnibus measure of DRV victimisation, emotional DRV victimisation and physical DRV victimisation.

An additional report(13) stratified DRV victimisation outcomes by ethnicity, comparing African-American and Latinx students separately without a formal test. There was no evidence of significant moderation, but effects for physical DRV victimisation were greater for African-American students and effects for emotional DRV victimisation were greater for Latinx students. In three short-term tests of omnibus measures of DRV victimisation, acculturation was not a significant moderator.

*Acculturation as a moderator of DRV victimisation outcomes*

One report(8) considered short-term moderation of DRV victimisation by acculturation in a sample of ethnic minority secondary school students. Two of three tests suggested numerically but not statistically greater effects for students with greater acculturation; the third was reported as non-significant only.

*Sexuality as a moderator of DRV victimisation outcomes*

The long-term evaluation of Green Dot(17) considered sexuality as a moderator of physical DRV victimisation and sexual DRV victimisation (specifically, reproductive coercion). While all tests were non-significant, six of eight tests suggested numerically but not statistically greater impacts for sexual majority students.

Sex as a moderator of DRV perpetration outcomes

Four evaluations provided some evidence of a gradient in effectiveness favouring boys. A long-term test of the impact of Fourth R(18) on physical DRV perpetration found evidence of a greater impact on boys (*p*=0.002). Three further evaluations found non-significant evidence of a greater impact on boys. In JOVEN, two longitudinal tests of emotional DRV perpetration and physical and sexual DRV perpetration found numerical but not statistical evidence of a greater impact on boys. Across eight long-term tests of emotional DRV perpetration and physical DRV perpetration from the evaluation of Green Dot,(1) five suggested numerically greater impact on boys as compared to girls. Nine short-term tests from the evaluation of Shifting Boundaries(15) included three tests of an omnibus measure of DRV perpetration, all of which were reported as non-significant only, and six tests of sexual DRV perpetration, of which four numerically favoured boys, one numerically favoured girls and one did not favour either group.

A further seven evaluations(3-6, 8-10, 12-14) did not provide evidence of a gradient in effectiveness by sex. One evaluation(13) included two long-term tests: one of physical DRV perpetration, which numerically favoured girls, and one of emotional DRV perpetration, which numerically favoured boys. The other six evaluations(3-6, 8-10, 12, 14) presented findings as non-significant only. The evaluation of Safe Dates(3-6) included 24 tests covering short-term,(3) long-term(4, 6) and longitudinal(5) outcomes for emotional DRV perpetration, physical DRV perpetration, and sexual DRV perpetration. The New York City evaluation of Fourth R(14) included one short-term and one long-term test of an omnibus measure of DRV perpetration. Two long-term tests from the evaluation of Teen Choices(10) included emotional DRV perpetration and physical DRV perpetration. The remaining three evaluations(8, 9, 12) presented short-term tests only: from the Katie Brown Educational Program,(9) five tests, four unique, covering an omnibus measure of DRV perpetration, physical DRV perpetration and emotional DRV perpetration; from another evaluation, two short-term tests of sexual DRV perpetration;(12) and finally, from one evaluation,(8) a test of an omnibus measure of DRV perpetration.

A final two evaluations(2, 19) provided short-term, non-significant evidence of a greater impact on girls. Across 24 tests (16 unique) including omnibus measure of DRV perpetration and sexual DRV perpetration, 21 tests indicated a numerically greater impact on girls, and three indicated a numerically greater impact on boys.(2) In another evaluation, intervention impacts on both emotional DRV perpetration and physical DRV perpetration showed numerically greater benefits for girls.

Prior history of the outcome as a moderator of DRV perpetration outcomes

Two reports provided some evidence of a gradient preferring those with prior history of DRV perpetration. Two long-term tests from the evaluation of Teen Choices(10) suggested numerically greater benefits of the intervention for those with prior history of DRV perpetration on physical DRV perpetration; these differences were significant for a test of emotional DRV perpetration. Nine short-term tests from the evaluation of Shifting Boundaries(15) related to omnibus measures of DRV perpetration and sexual DRV perpetration. None of the tests were significant. Three of the tests were reported as non-significant only, four were reported as showing numerically greater benefits for those with prior history of DRV perpetration, and two showed numerically greater benefits for those without a prior history of DRV perpetration. Finally, evidence from Safe Dates included short-term,(3) long-term(4, 6) and longitudinal(5) tests relating to emotional DRV perpetration, physical DRV perpetration, and sexual DRV perpetration. Only two(4, 5) of the four reports included formal tests. In the two reports(3, 6) that did not include formal tests, there was no clear evidence of significant moderation, though three of eight tests numerically preferred those with no prior history and five of eight tests numerically preferred those with prior history. In long-term and longitudinal evidence (16 tests), three tests were significant and indicated greater benefits for those with no prior history. The remaining 13 tests were reported as non-significant only.

Dating history as a moderator of DRV perpetration outcomes

Two outcome evaluations(3, 6, 16) considered dating history as a moderator of DRV perpetration. None of the reports presented formal tests. In short-term(3) and long-term(6) evidence from Safe Dates, eight tests covered physical DRV perpetration, sexual DRV perpetration and emotional DRV perpetration. Numerical estimates for the dating subsample were similar to estimates for the entire population across all tests, suggesting no significant moderation; however, estimates numerically favoured those with dating history in five tests and did not favour either group in another test. In long-term evidence from another evaluation,(16) six tests (five unique) of physical DRV perpetration, sexual DRV perpetration, emotional DRV perpetration and cyber DRV perpetration, as well an omnibus measure of DRV perpetration, were presented. Evidence was equivocal, as comparing estimates for those with dating history to the entire population suggested little difference between groups. Numerically, three tests favoured those with dating history and three tests favoured those without dating history, though it was unlikely any difference was statistically significant.

*Age as a moderator of DRV perpetration outcomes*

The long-term evaluation(10) of Teen Choices evaluated grade level as a moderator of effects on physical DRV perpetration and emotional DRV perpetration. In both cases, results were reported as non-significant only.

*Ethnicity as a moderator of DRV perpetration outcomes*

Three outcome evaluations(4-6, 9, 10) considered ethnicity (White vs other) as a moderator of DRV perpetration outcomes. All reported findings as non-significant only. This included long-term(4, 6) and longitudinal(5) evidence from Safe Dates over 20 tests covering physical DRV perpetration, emotional DRV perpetration and sexual DRV perpetration; two long-term tests from Teen Choices(10) covering sexual DRV perpetration and emotional DRV perpetration; and five short-term tests (four unique) from the Katie Brown Educational Program(9) covering emotional DRV perpetration, physical DRV perpetration and an omnibus measure of DRV perpetration.

An additional report(13) stratified DRV perpetration outcomes by ethnicity, comparing African-American and Latinx students separately without a formal test. There was no evidence of significant moderation, but effects for physical DRV perpetration were greater for African-American students and effects for emotional DRV perpetration were greater for Latinx students.

*Acculturation as a moderator of DRV perpetration outcomes*

One study(8) considered moderation of a short-term omnibus measure of DRV perpetration by level of acculturation. This was reported as non-significant only.

*Sexuality as a moderator of DRV perpetration outcomes*

The long-term evaluation of Green Dot(17) considered sexuality as a moderator of physical DRV perpetration over four tests. Two tests were non-significant but numerically favoured sexual minority students; one test was non-significant but numerically favoured sexual majority students; and one test generated significant evidence of greater impact on sexual majority students.

Sex as a moderator of GBV victimisation outcomes

Four outcome evaluations(2, 20-22) suggested a greater impact for boys. One report of an intervention in South Africa(20) considered physical GBV victimisation (specifically, forced sex) over short-term and long-term timeframes. All six tests indicated a greater impact on boys than girls, with three generating a significant result. Long-term evidence from an outcome evaluation in India(22) included four relevant tests of an omnibus measure of GBV. Three of four tests favoured boys, and two of these were imputed as significant. Long-term evidence from an outcome evaluation of the Good Schools Toolkit(21) included three tests of an omnibus measure of GBV victimisation. All were non-significant, but all three numerically favoured boys. Lastly, short-term evidence from the Cleveland evaluation of interaction-based and law and justice-based interventions(2) generated eight tests of moderation by sex. All were non-significant, but five numerically favoured boys.

A further five evaluations(1, 12, 14, 23, 24) did not offer evidence of a gradient in effectiveness by sex. The evaluation of Green Dot provided long-term evidence of moderation by sex over 24 tests relating to physical GBV victimisation and verbal GBV victimisation, 20 of which were unique. While all were non-significant, of the 20 unique tests, two demonstrated no numerical difference between groups, nine suggested a numerical benefit for boys, and nine suggested a numerical benefit for girls. Of the remaining four non-unique tests, three favoured girls, though all were non-significant. A subsequent four evaluations(12, 14, 23, 24) only presented evidence of non-significance. The New York City evaluation of Fourth R(14) included short-term and long-term tests of an omnibus measure of GBV victimisation. Another evaluation(23) presented four long-term tests, of which two were of an omnibus measure of GBV victimisation and two were of verbal GBV victimisation. In the remaining two evaluations, all tests were short-term only. One evaluation(24) reported two tests of an omnibus measure of GBV victimisation, and one evaluation(12) reported two tests of physical GBV victimisation.

Finally, two evaluations suggested a greater impact on girls. One outcome evaluation(22) in Vietnam presented three long-term tests (two unique) of omnibus measures of GBV victimisation and of physical GBV victimisation. While all three tests numerically favoured girls, two were imputed as being statistically significant. Short-term tests from the New York City evaluation of Shifting Boundaries(15) included six tests of an omnibus measure of GBV victimisation. All were non-significant, but five of six yielded numerically greater benefits for girls.

*Prior history of the outcome as a moderator of GBV victimisation outcomes*

Three evaluations(14, 15, 25) considered prior history of GBV victimisation as a moderator of intervention outcomes. Two evaluations were largely equivocal. The evaluation of Shifting Boundaries(15) included six tests of an omnibus measure of GBV victimisation, all short-term. While all tests were non-significant, three tests numerically favoured those with no prior history of GBV victimisation and three tests numerically favoured those with prior history. The New York City evaluation of Fourth R(14) included short-term and long-term tests of an omnibus measure of GBV victimisation. While both were non-significant, the short-term test numerically favoured those with prior history of GBV victimisation and the long-term test numerically favoured those with no prior history of the outcome. A final evaluation was delivered only to girls and included two short-term tests of physical GBV victimisation and one short-term test of verbal GBV victimisation. Both tests of physical GBV victimisation were non-significant but indicated greater impact for those with prior history of GBV victimisation, while the test verbal GBV victimisation was significant and indicated greater impacts for those with prior history.

*Age as a moderator of GBV victimisation outcomes*

Three outcome evaluations(23, 24, 26) tested age as a moderator of GBV victimisation outcomes. One evaluation(24) considered two short-term omnibus measures of GBV victimisation by grade level and reported the moderation test as non-significant only. Another evaluation considered two short-term measures of physical GBV victimisation by school stage; while both tests were non-significant, one favoured younger students and the other favoured older students. A final evaluation(23) presented four long-term tests, of which two were of an omnibus measure of GBV victimisation and two were of verbal GBV perpetration victimisation. All were reported as non-significant only.

*Ethnicity as a moderator of GBV victimisation outcomes*

Two outcome evaluations(23, 24) tested ethnicity (White vs other) as a moderator of GBV victimisation outcomes. One evaluation(24) considered two short-term omnibus measures of GBV victimisation and reported the moderation tests as non-significant only. Another evaluation(23) presented four long-term tests, of which two were of an omnibus measure of GBV victimisation and two were of verbal GBV victimisation. All tests were reported as non-significant only.

*Sexuality as a moderator of GBV victimisation outcomes*

Two outcome evaluations(17, 23) tested sexuality as a moderator of GBV victimisation outcomes. Both considered long-term effects only. The evaluation of Green Dot(17) tested sexuality as a moderator of physical GBV victimisation and verbal GBV victimisation for a total of 12 tests. Eight of 12 tests demonstrated numerically greater benefits for sexual majority students; of these, one test of a verbal GBV victimisation outcome was statistically significant. Another evaluation(23) presented four long-term tests, of which two were of an omnibus measure of GBV victimisation and two were of verbal GBV victimisation. All tests were reported as non-significant only.

*Poverty status as a moderator of GBV victimisation outcomes*

One evaluation(23) presented four long-term tests, of which two were of an omnibus measure of GBV victimisation and two were of verbal GBV victimisation. All tests were reported as non-significant only.

*Age as a moderator of GBV perpetration outcomes*

Two outcome evaluations(23, 24) tested age as a moderator of GBV perpetration outcomes. One evaluation(24) considered a short-term omnibus measure of GBV perpetration and reported the moderation test as non-significant only. Another evaluation(23) presented four long-term tests, of which two were of an omnibus measure of GBV perpetration and two were of verbal GBV perpetration. Both tests of the omnibus measure were reported as non-significant only; one test of verbal GBV perpetration was reported as non-significant only, whereas one test of verbal GBV perpetration reported a significant result favouring greater impacts for younger students.

*Ethnicity as a moderator of GBV perpetration outcomes*

Two outcome evaluations(23, 24) tested ethnicity (White vs other) as a moderator of GBV perpetration outcomes. One evaluation(24) considered a short-term omnibus measure of GBV perpetration and reported the moderation test as non-significant only. Another evaluation(23) presented four long-term tests, of which two were of an omnibus measure of GBV perpetration and two were of verbal GBV perpetration. All tests were reported as non-significant only.

*Sexuality as a moderator of GBV perpetration outcomes*

Two outcome evaluations(17, 23) tested sexuality as a moderator of GBV perpetration outcomes. Both considered long-term effects only. The evaluation of Green Dot(17) tested sexuality as a moderator of physical GBV perpetration and verbal GBV perpetration for a total of 12 tests. None were statistically significant, but eight of 12 tests demonstrated numerically greater benefits for sexual majority students. Another evaluation(23) presented four long-term tests, of which two were of an omnibus measure of GBV perpetration and two were of verbal GBV perpetration. Three tests were reported as non-significant only; one test of an omnibus measure of GBV perpetration demonstrated statistically greater benefits for heterosexual students.

*Poverty status as a moderator of GBV perpetration outcomes*

One evaluation(23) presented four long-term tests, of which two were of an omnibus measure of GBV perpetration and two were of verbal GBV perpetration. All tests were reported as non-significant only.

# References

1. Coker AL, Bush HM, Cook-Craig PG, DeGue SA, Clear ER, Brancato CJ, et al. RCT testing bystander effectiveness to reduce violence. American Journal of Preventive Medicine. 2017;52(5):566-78.

2. Taylor BG, Stein N, Burden FF. Exploring gender differences in dating violence/harassment prevention programming in middle schools: results from a randomized experiment. Journal of Experimental Criminology. 2010;6(4):419-45.

3. Foshee VA, Bauman KE, Arriaga XB, Helms RW, Koch GG, Linder GF. An evaluation of Safe Dates, an adolescent dating violence prevention program. American Journal of Public Health. 1998;88(1):45-50.

4. Foshee VA, Bauman KE, Ennett ST, Linder GF, Benefield T, Suchindran C. Assessing the long-term effects of the Safe Dates program and a booster in preventing and reducing adolescent dating violence victimization and perpetration. American Journal of Public Health. 2004;94(4):619-24.

5. Foshee VA, Bauman KE, Ennett ST, Suchindran C, Benefield T, Linder GF. Assessing the effects of the dating violence prevention program "Safe Dates" using random coefficient regression modeling. Prevention Science. 2005;6(3):245-58.

6. Foshee VA, Bauman KE, Greene WF, Koch GG, Linder GF, MacDougall JE. The Safe Dates program: 1-year follow-up results. American Journal of Public Health. 2000;90(10):1619-22.

7. Gonzalez-Guarda RM, Guerra JE, Cummings AA, Pino K, Becerra MM. Examining the preliminary efficacy of a dating violence prevention program for Hispanic adolescents. Journal of School Nursing. 2015;31(6):411-21.

8. Jaycox LH, McCaffrey D, Eiseman B, Aronoff J, Shelley GA, Collins RL, et al. Impact of a school-based dating violence prevention program among Latino teens: randomized controlled effectiveness trial. Journal of Adolescent Health. 2006;39(5):694-704.

9. Joppa MC, Rizzo CJ, Nieves AV, Brown LK. Pilot investigation of the Katie Brown Educational Program: A school-community partnership. Journal of School Health. 2016;86(4):288-97.

10. Levesque DA, Johnson JL, Welch CA, Prochaska JM, Paiva AL. Teen dating violence prevention: Cluster-randomized trial of teen choices, an online, stage-based program for healthy, nonviolent relationships. Psychology of Violence. 2016;6(3):421-32.

11. Miller E, Goldstein S, McCauley HL, Jones KA, Dick RN, Jetton J, et al. A school health center intervention for abusive adolescent relationships: a cluster RCT. Pediatrics. 2015;135(1):76-85.

12. Muck C, Schiller EM, Zimmermann M, Kartner J. Preventing sexual violence in adolescence: Comparison of a scientist-practitioner program and a practitioner program using a cluster-randomized design. Journal of Interpersonal Violence. 2018;36(3-4):NP1913-40NP.

13. Peskin MF, Markham CM, Shegog R, Baumler ER, Addy RC, Tortolero SR. Effects of the It's Your Game . . . Keep It Real program on dating violence in ethnic-minority middle school youths: a group randomized trial. American Journal of Public Health. 2014;104(8):1471-7.

14. Cissner AB, Ayoub LH. Building healthy teen relationships: An evaluation of the Fourth R Curriculum with middle school students in the Bronx: U.S. Washington, DC: Center for Court Innovation; 2014.

15. Taylor BG, Mumford EA, Stein ND. Effectiveness of "Shifting Boundaries" teen dating violence prevention program for subgroups of middle school students. Journal of Adolescent Health. 2015;56(2 Suppl 2):S20-6.

16. Peskin MF, Markham CM, Shegog R, Baumler ER, Addy RC, Temple JR, et al. Adolescent dating violence prevention program for early adolescents: The Me & You randomized controlled trial, 2014-2015. American Journal of Public Health. 2019;109(10):1419-28.

17. Coker AL, Bush HM, Clear ER, Brancato CJ, McCauley HL. Bystander program effectiveness to reduce violence and violence acceptance within sexual minority male and female high school students using a cluster RCT. Prevention Science. 2020;21(3):434-44.

18. Wolfe DA, Crooks C, Jaffe P, Chiodo D, Hughes R, Ellis W, et al. A school-based program to prevent adolescent dating violence: a cluster randomized trial. Archives of Pediatrics and Adolescent Medicine. 2009;163(8):692-9.

19. Munoz-Rivas MJ, Redondo-Rodriguez N, Ronzon-Tirado RC. Dating violence prevention: Evaluation of the program PREVIO. Revista de Psicologia Clinica con Niños y Adolescentes. 2019;6(3):18-23.

20. Jemmott JB, 3rd, O'Leary A, Jemmott LS, Ngwane ZP, Teitelman AM, Makiwane MB, et al. Effect of a behavioral intervention on perpetrating and experiencing forced sex among South African adolescents: A secondary analysis of a cluster randomized trial. JAMA Network Open. 2018;1(4):e181213.

21. Devries KM, Knight L, Allen E, Parkes J, Kyegombe N, Naker D. Does the Good Schools Toolkit reduce physical, sexual and emotional violence, and injuries, in girls and boys equally? A cluster-randomised controlled trial. Prevention Science. 2017;18(7):839-53.

22. ICRW. Changing course. Implementation and evaluation of the Gender Equity Movement in Schools (GEMS) program in specific sites - Vietnam, India and Bangladesh. New Delhi, India: International Center for Research on Women; 2017.

23. Waterman EA, Edwards KM, Banyard VL, Chang H. Age and sexual orientation moderated the effects of a bystander-focused interpersonal violence prevention program for high school students. Prevention Science. 2021.

24. de Lijster GP, Felten H, Kok G, Kocken PL. Effects of an interactive school-based program for preventing adolescent sexual harassment: A cluster-randomized controlled evaluation study. Journal of Youth and Adolescence. 2016;45(5):874-86.

25. Rowe LS, Jouriles EN, McDonald R. Reducing sexual victimization among adolescent girls: a randomized controlled pilot trial of My Voice, My Choice. Behavior Therapy. 2015;46(3):315-27.

26. Decker MR, Wood SN, Ndinda E, Yenokyan G, Sinclair J, Maksud N, et al. Sexual violence among adolescent girls and young women in Malawi: A cluster-randomized controlled implementation trial of empowerment self-defense training. BMC Public Health. 2018;18(1):1341.
